# Draft genome sequences of multidrug-resistant *Escherichia coli* strains from the human gut

**DOI:** 10.1128/mra.00479-25

**Published:** 2025-07-31

**Authors:** Misbah Tubassam, Zumara Younus, Rida Habib, Muhammad Imran

**Affiliations:** 1 Department of Microbiology, Faculty of Biological Sciences, Quaid-i-Azam University 429798, Islamabad, Pakistan; University of Maryland School of Medicine, Baltimore, Maryland, USA

**Keywords:** human gut microbiome, *E. coli*, draft genome

## Abstract

Draft genome sequences of multidrug-resistant *Escherichia coli* strains isolated from human gut are reported here. The size of the draft genome ranged from 4,985,384 to 5,220,996 bp. Genome annotation predicted 3,731–3,909 protein-coding genes while 47–60 antimicrobial resistance genes.

## ANNOUNCEMENT

*Escherichia coli*, a predominant gram-negative gut commensal, has emerged as a significant antimicrobial-resistant (AMR) threat due to its ability to acquire and transfer resistance genes (1), which not only enhances its own survival but also accelerates the spread of AMR to other pathogenic bacteria, emphasizing the need for genomic surveillance (2, 3).

Ethical approval for the collection and use of human stool samples was obtained from the Bio-Ethical Committee (BEC) of Quaid-I-Azam University, Islamabad, Pakistan, under protocol number (**BEC-FBS-QAU2018-115**), as well as consent was taken from participants. Human stool samples were collected aseptically in sterile containers with phosphate buffer saline, stored at −20°C and enriched in Tetrathionate Broth at 37°C for 24 hrs. Culturing was performed on MacConkey agar plates and colonies were purified based on their appearance, lactose fermentation (pink/red colonies), and Gram staining. Biochemical confirmation was performed using the API 10S kit (BioMérieux) (4–6). Purified isolates were cultured in Tryptic Soy Broth at 37°C for 18 h under aerobic conditions. Genomic DNA was extracted using the FavorPrep Stool DNA Isolation Mini Kit (Favorgen, Taiwan) and integrity was assessed via 1% agarose gel electrophoresis (7). The whole genome of strains was sequenced with Oxford Nanopore Technologies MinION platform using a ligation-based protocol (SQK-LSK109, R9.4 flow cell). DNA was not sheared or size-selected prior to library preparation. Real-time base calling was performed using Guppy Basecaller (v.3.6.0) (8), basecalled reads N50 (Table 1) were calculated with NanoPlot (v1.28.2) (9). Read quality was assessed using FastQC (v.0.12.0) (10) and processed through the ONT EPI2Me (WGS) workflow for assembly. The completeness and accuracy of the assembled genome were evaluated using CheckM (version 1.2.2) (11). Assemblies were annotated by using the NCBI Prokaryotic Genome Annotation Pipeline (PGAP, v.5.0) (12). All the draft genome information and results are presented in Table 1. All tools were used with default parameters unless otherwise specified.

**TABLE 1:** Genome statistics for *E. coli* isolates obtained from human gut

Feature	*E. coli* zumi 6	*E. coli* zumi 8
Bio Project	PRJNA681996	PRJNA681996
Bio sample	SAMN16976420	SAMN16976422
GenBank	JAEAGI000000000	JAEAGH000000000
Version	JAEAGI000000000.1	JAEAGH000000000.1
WGS	JAEAGI010000001-JAEAGI010000002	JAEAGH010000001-JAEAGH010000004
SRA	SRR33296409	SRR33296408
Genome size	5,220,996	4,985,384
GC content	50.4	50.8
Read N50	5,012.0	7,198.0
Contigs N50	5,107,798	4,804,817
Number of contigs	2	4
Genes (total)	4,994	4,816
CDSs (total)	4,881	4,694
Number of genes (Coding)	3,909	3,731
CDSs (with protein)	3,909	3,731
Number of genes (RNA)	113	122
rRNAs	8 (5S), 7 (16S), 7 (23S)	8 (5S), 7 (16S), 7 (23S)
tRNAs	85	87
Number of subsystems	601	604
Pseudo genes	972	963
CRISPR arrays		2
AMR	43	60
AMR genes	(*aadA*, *aph(3")-Ib*, *aph(6)-Id*), β-lactams (*blaTEM-1*), tetracyclines (*tet(A*)), sulfonamides (*sul1*, *sul2*), trimethoprim (*dfrA*), and fluoroquinolones (*qnrS1*)	β-lactams *blaTEM-1*, sulfonamides *sul1*, tetracyclines (*tet(A*)), *aadA*, fluoroquinolones (*qnrS1*), chloramphenicol (*catB*)

Phenotypic antimicrobial susceptibility testing was performed by the Kirby–Bauer disk diffusion method in accordance with CLSI guidelines (13, 14). Both strains exhibited multidrug-resistance to β-lactams, aminoglycosides, fluoroquinolones, tetracyclines, and sulfonamides. Resistance gene profiling using the Comprehensive Antibiotic Resistance Database (v6.0.2) (15) confirmed the presence of multiple AMR determinants consistent with phenotypic resistance (Table 1).

Further comparative genomic analysis and detailed whole genome analysis, necessary to confirm the genetic context and mobility of these resistance determinants, will be presented in future publication. This genomic resource announcement provides valuable insights into the genetic basis of AMR in gut resident *E. coli* and its implications for public health.

## Data Availability

The genomic data reported here has been deposited in GenBank under the accession numbers JAEAGI010000001-JAEAGI010000002 and JAEAGH010000001-JAEAGH010000004. The Bio Project number is PRJNA681996, and the relevant BioSample numbers are SAMN16976420 to SAMN16976422 . Raw sequencing data has been deposited in SRA under the accession nmbers SRR33296409 and SRR33296408.
